# Quality of Life Outcomes According to Differential Nusinersen Exposure in Pediatric Spinal Muscular Atrophy

**DOI:** 10.3390/children8070604

**Published:** 2021-07-17

**Authors:** Meaghann S. Weaver, Alice Yuroff, Sarah Sund, Scott Hetzel, Matthew A. Halanski

**Affiliations:** 1 Children’s Hospital and Medical Center, Omaha, NE 68114, USA; meweaver@childrensomaha.org; 2 National Center for Ethics in Healthcare, Washington, DC 20420, USA; 3 School of Medicine and Public Health, University of Wisconsin-Madison, Madison, WI 53706, USA; Alice.Yuroff@fammed.wisc.edu (A.Y.); Sund@ortho.wisc.edu (S.S.); hetzel@biostat.wisc.edu (S.H.)

**Keywords:** spinal muscular atrophy, quality of life, child neurology, patient-reported outcomes, neuromuscular

## Abstract

The purpose of this study was to explore early changes in patient and family caregiver report of quality of life and family impact during the transitional period of nusinersen use. Communication; family relationships; physical, emotional, social, and cognitive functioning; and daily activities were measured using Pediatric Quality of Life modules (Family Impact Modules and both Patient and Proxy Neuromuscular-Specific Reports) pre- and post-nusinersen exposure. A total of 35 patients with SMA (15 Type 1, 14 Type 2, and 6 Type 3) were grouped according to nusinersen exposure. When analyzed as a whole cross-sectional clinical population, no significant differences were found between the initial and final surveys. Nusinersen therapy was associated with improved communication and emotional functioning in subsets of the population, particularly for patients on maintenance therapy for longer duration. Several unexpected potentially negative findings including increases in family resources and trends towards increases in worry warrant further consideration. Further research is warranted to explore the impact of novel pharmaceuticals on quality of life for children with SMA longitudinally to optimize clinical and psychosocial outcomes.

## 1. Introduction

Spinal muscular atrophy (SMA) is an autosomal-recessive, progressive neuromuscular disease associated with extensive morbidity related to muscular atrophy and proximal muscle weakness with risk for early mortality. In the past, children with SMA Type I seldom survived beyond the first few years of life even with mechanical respiratory support [1]. With the recent introduction of novel pharmaceutical interventions such as nusinersen [1,2] and gene therapies [3], children with SMA now have potentially increased lifespans and improved quality of life (QOL). A modified 2’-O-methoxyethyl antisense oligonucleotide by the name of nusinersen was approved by the Food and Drug Agency in December of 2016 with subsequent evidence of high efficacy and safety [4,5]. Quality of life outcomes associated with nusinersen use have been less studied.

A paucity of data exists on how children with SMA depict quality of life from their own report or how family caregivers of children with SMA perceive the diagnosis impacts the child and family before, during, and after the early phases of introducing nusinersen. As survival may be prolonged through medical advancements, learning about the child’s QOL remains a compassionate, competent clinical care priority [6,7]. This knowledge can help clinicians partner with the child and family for symptom or support interventions intended to further support lived experiences. QOL is defined as “an individual’s perception of his/her position in life in the context of culture and value systems in which he/she lives and in relation to wellness, goals, expectations, standards, and concerns” [8]. By investing in the subjective perspective of pediatric patients and their family caregivers before and after introduction of a therapy such as nusinersen, clinical teams are then positioned to better appreciate how therapies may trend with enhanced or burdened overall perceptions of health or wellness.

In 2016, prior to the widespread use of nusinersen at our institution, we began a study to evaluate the sensitivity of the Caregiver Priorities and Child Health Index of Life with Disabilities (CPCHILD™) questionnaire and PedsQL™ 3.0 Neuromuscular Module NMM (PedsQL) outcome measures to detect uniqueness between the between patient and proxy measurements and between SMA types [9]. We extended collection of the PedsQL outcome measures through 2019 to detect early changes in PEdsQL measurements in our clinical population during the transitional period of nusinersen use. This study highlights the early differences in PedsQL according to nusinersen exposure. 

## 2. Materials and Methods

### 2.1. Participants and Setting

The University of Wisconsin-Madison Minimal Risk Institutional Review Board approved the study methodology and the ethics of implementation of this health sciences study in October 2015. Children and their family caregiver proxy were enrolled from November 2016 to September 2019. Eligibility criteria included patients with a diagnosis of SMA currently younger than age 18 receiving care at the outpatient neuromuscular clinic. 

A letter was sent to eligible children/families providing details about the voluntary research study opportunity. The letter was mailed one to two weeks prior to the eligible subject’s scheduled outpatient visit. The study coordinator then offered to meet with the patient and family caregiver through an informed consent process at the clinic visit. iPads linked wirelessly to the RedCAP© study database served as the survey response collection modality. The initial study included cross-over assessment of both the PedsQL™ 3.0 Neuromuscular Module (NMM) (for child-report and proxy-report) coupled with the PedsQL™ Family Impact Module (FIM) or CPCHILD™ questionnaire [9]. We continued collection of outcome measures until 2019 using the PedsQL measures as these appeared the most sensitive in our previous study in this population [9].

**Figure 1:**
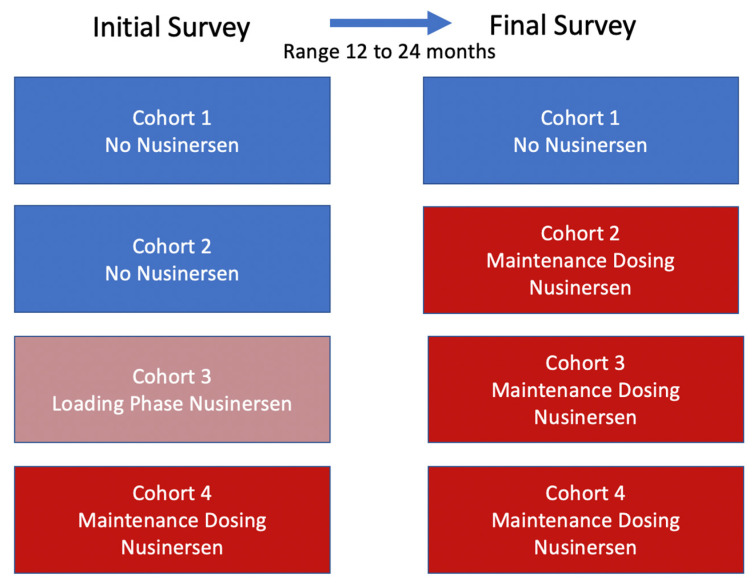
Nusinersen exposure at initial and final study timepoints. Legend—Transitions between nusinersen exposure during study initiation and follow-up timepoint including Cohort 1 which remained nusinersen naïve throughout; Cohort 2 which transitioned from no nusinersen to maintenance dosing; Cohort 3 which transitioned from loading phase to maintenance dosing; and Cohort 4 which remained on maintenance dosing with longest steady exposure to nusinersen.

The electronic medical record was reviewed in a retrospective nature to determine the nusinersen status at the time of each initial questionnaire to produce four cohorts: (1) No Intent of Treatment, which included patients who did not start nor proceeded with any nusinesen treatment at time of final outcome measure; (2) Intent of Treatment, which included patients not on any treatment at time of initial questionnaire but began therapy after the initial assessment; (3) Loading Phase, which included patients within the first two months of treatment (received four intrathecal infusions) at the first quality of life (QOL) assessment; and (4) Maintenance Phase, which included patients on maintenance schedule (infusion every 4 months) at the time of initial QOL assessment. Cohorts 2–4 were receiving maintenance dosing at the time of their final survey (Figure 1). Pairwise differences between initial and final scores were analyzed within each cohort.

### 2.2. Methods

The PedsQL 3.0 Neuromuscular Module (NMM) includes 25 items covering core dimensions: (1) About My Neuromuscular Disease (17 items with emphasis on physical functioning), (2) Communication (3 items), and (3) About Our Family Resources (5 items). Child self-report and family proxy-reports are summarized for the past month. The PedsQL™ NMM maintains Cronbach’s coefficient alpha scores >0.77 for each scale dimension in SMA cohorts [10,11,12].

The 36 item PedsQL Family Impact Module (FIM) measures parental perceptions of parental self-reported physical functioning (6 items), emotional functioning (5 items), social functioning (4 items), cognitive functioning (5 items), communication (3 items), and worry (5 items). The parent is reporting on his/her own well-being rather than the child’s well-being over the past month. The PedsQL FIM explores the impact of the child’s SMA diagnosis and neuromuscular health on family daily activities (3 items) and family relationships (5 items). In validation studies, Cronbach’s coefficient alpha scores were >0.82 for PedsQL FIM scales [13].

### 2.3. Statistical Analysis

Survey total scores and sub-scores were calculated. Results were summarized using mean (SD) at each timepoint and the difference of means between the initial and final survey reported. Separate analyses were then performed based on SMA type or initial nusinersen status for those receiving nusinersen. Subjects in Cohorts 2 and 3 (no nusinersen at baseline and those in the loading phase) were also pooled together to assess any changes occurring once maintenance dosing is achieved. This combined cohort was then compared with those at on maintenance dosing at the time of initial survey. Comparisons of survey scores across two-level factors utilized t-tests, while comparisons of survey scores across three-level factors utilized ANOVA models. Due to the magnitude of testing, *p*-values were Benjamini–Hochberg corrected to control for false discovery rate [14]. Significant ANOVA *p*-values resulted in post hoc pairwise t-tests with Holm-adjusted *p*-values [15]. All tests had an adjusted alpha level of 0.05 and were conducted using R for Statistical Computing Version 3.5 [16].

## 3. Results

### 3.1. Participants

A total of 35 patients with SMA: 15 Type 1, 14 Type 2, and 6 Type 3, with a respective average age at initial survey (2.7+/−2.1), (11.2+/−5.9), (10.2+/−2.7) years and an average 1.8 (+/−0.5) years between surveys were analyzed. Five patients were in Cohort 1, the non-treatment control cohort containing two patients with Type 1, one patient with Type 2, and two patients with Type 3. Cohort 2 had *n =* 8 that had not started nusinersen at the time of the first survey, Cohort 3 had *n =* 11 that were in the loading phase of nusinsersen at the time of the first survey, and Cohort 4 had *n* *=* 11 subjects already at the maintenance phase of treatment at the time of initial PedsQL. All patients in Cohorts 2–4 were on maintenance dosing at the time of the final QOL survey.

### 3.2. Collective Cohort

When analyzed as a whole cross-sectional clinical population (pooling Cohorts 2–4), no significant differences were found between the initial and final surveys for family impact (Table 1), child self-report (Table 2), or proxy family caregiver report (Table 3).

After sub-analyzing the data by SMA type and cohort (nusinersen status at the initial survey), several significant differences and trends were identified (Table 4 and Table 5). In the Family Impact Module, improvements in emotional functioning were observed for children (*n* = 8) that progressed from no treatment to maintenance therapy (56.2+/−7.5→65.4+/−15.3, *p* = 0.014). 

Patients on the maintenance dosing at the time of the initial questionnaire and therefore on maintenance therapy for longer duration (increased time exposure to nusinersen) demonstrated significant improvements in communication (43.3+/−19.6→54.2+/−25, *p* = 0.041). Per the Parental-PedsQL, improvements in communication trended towards improvement in patients initiating therapy and reaching maintenance dosing (Cohort 2) (53.1 +/−34.5→67.7+/−32.6, *p* = 0.089) and became significant when pooled with Cohort 3 (45.2+/−34.5→53.9+/−33.7, *p* = 0.04) demonstrating an improvement in communication scores when maintenance dosing was achieved and sustained. 

Improvements in daily activities (39.1+/−28.1→52.6+/−32.5, *p* = 0.065) and PedsQL Family Impact Total Score (59.2+/−21.2→64.2+/−21.8, *p* = 0.081) domains trended towards significance in the population with SMA Type 2. 

Patients on the maintenance dosing at the time of the initial questionnaire (again, with increased time exposure to nusinersen) demonstrated significant improvements in PedsQL Family Impact Total Score (45.7+/−13→52.6+/−26.1; *p* = 0.027).

While the majority of findings demonstrated improvements for the patents and families undergoing nusinersen treatment, several unaccepted adverse findings became apparent. First, the Family Resources Domain in the Child-PedsQL (N = 4) was significantly higher at follow up in the SMA Type 1 cohort (36.7+/−15.3→71.2+/−31.7, *p* = 0.003) and appeared to most affect those progressing from the loading to the maintenance phase (52.5+/−26→71.7+/−15.4; *p =* 0.015) (N = 7) and trended similarly in the Parental-PedsQL for patients with SMA Type 3 (58.8+/−12.5→68.8+/−15.5, *p* = 0.066).

Increases in the Worry domain also trended towards significance in our Family Impact Module for the SMA Type 1 cohort (45+/−12.7→50.8+/−12.6; *p =* 0.054) and surprisingly for those on the maintenance dosing for the entirety of this study (Cohort 4) 44.5+/−16.1→57.9+/−23.2, *p =* 0.056.

## 4. Discussion

In the PedsQL scale, a change of 5 in the Standard Error of the Mean (SEM) has been pre-determined to represent a minimally clinically important difference [17,18]. Thus, while noted change did not reach statistical significant difference when the at-large group was analyzed, MCID was reached for communication and family resources according to child self-report. 

The major domains impacted during nusinersen treatment between the no treatment cohort and all SMA types were communication and emotional functioning. The major domain impacted during nusinersen treatment between SMA Type 1 and other SMA types was worry. Of interest, improvements in daily activities and Family Impact total score were significant only in patients with SMA Type 2. This may be due to the number of patients with Type 2 included in this study or may be due to other factors since there was no difference observed in baseline and follow up. Prior analyses by SMA type have revealed benefits in axial, proximal, and distal motor function, particularly for those with more severe forms of the disorder [3,4].

In this cross-sectional clinical study, utilizing patient reported outcome measures validated in the SMA population, nusinersen therapy was found to improve communication and emotional functioning in subsets of the population. However, several unexpected potentially negative findings including increases in family resources and trends towards increases in worry (particularly in those on the medication for the longest period of time) warrant further consideration.

### 4.1. Improvement in Communication and Emotional Functioning

This study revealed the benefits of nusinersen on psychosocial function beyond physiologic metrics, recognizing the importance of family communication for starting the medication and in goal setting for sustaining the medication. Nusinersen has been shown to prolong survival in infants with SMA [19,20,21,22] and improve motor function [23,24] and yet the impact of treatment options on family-based communication quality and satisfaction has been under-explored. The PedsQL FIM specifically asks about the experience of the family in communicating with the child’s doctors and nurses about how they feel in addition to questions about communicating with friends and other extended family members. In a qualitative study of 19 parents engaged in decision making for their children with SMA, the most important factor for parental decision making was “honest communication with physicians” [25]. For parents in Germany whose children received nusinersen via an expanded access program, “good communication and trusting relationships with medical and non-medical staff at the hospital helped caregivers cope with the uncertainties associated with the treatment” [26]. Fifty-one parents of Swedish children with SMA emphasized the desire for health care professionals to not only possess knowledge but to provide knowledge [27], seemingly as a means to foster family communication and concordance in family decision making. 

A population-based study among 34 Danish parents of children with severe SMA revealed the prioritized importance parents place on provider communication that specifies what SMA entails, the treatment options, and prognosis [28]. Among 95 parents of children with severe SMA in Denmark and Sweden, bereaved parents were significantly more satisfied with care than non-bereaved parents (81% vs. 29%), with noted emphasis on communication as part of care coordination [29].

While medical outcomes matter, families also highly regard and uphold the process of communication as formative in their family experience. Introducing nusinersen as a treatment option necessarily results in engagement about current and anticipated research findings, potential benefits and harms, and experiences of other families. This treatment-dialogue has potential to improve knowledge and empower communication within families.

### 4.2. Increase in Use of Family Resources

This study revealed that use of family resources was perceived as significantly increased for children with Type 1 SMA receiving nusinersen according to child self-report. Parents in this study did not document parallel perception of increased use of family resources according. This speaks to the pediatric patient’s awareness of the investment of family time and finances to nusinersen as a biomedical intervention. The ways in which children with complex care needs may internally compare their resource requirements as compared to healthy peers or siblings, and how this translates into a child’s sense of self (whether the child views herself as worthy or as burdensome) or perception of stress (whether the child carries undue fear about fiscal wellness for others in the family) have been under-explored and even under-recognized by health systems. Parents in this study may have normalized resource utilization out of deep regard for their child’s access to the intervention and inability to place a resource measure on the infinite value of their child’s life. 

Parents of children starting nusinersen describe striving for longer duration of life and improved quality of life [30] in the setting of invasive treatment and complex care with frequent hospital-based procedures. Parents of children with SMA starting nusinersen have reported worries about the high cost and maintaining adequate insurance coverage; potential side effects, risk factors, and adverse events; and treatment time [31]. The indirect care coverage costs and foregone parental employment add to the direct medical costs along with the hidden cost of mental health strain [32]. In a study of 64 parents of children with SMA, family finances were depicted as an under-recognized and yet realistic family concern [33]. In a study of parents of children w/ SMA Types 2 and 3 in Australia, parents described: “significant financial and caregiving burdens, adjusted career choices and limitations on career progression and a complex landscape of access to funding, equipment, support and resources” [32]. 

From a health system perspective, the average annual cost of SMA1 “ranged from $75,047 to $196,429 per year” [34]. The “incremental cost-effectiveness ratio (ICER) of nusinersen compared to standard of care in SMA1 ranged from $210,095 to $1,150,455 per quality-adjusted life years (QALY) gained.” [34] In a health resource comparison study, patients in the SMA Type I group (*n =* 349) and SMA Type 1 nusineran group (*n =* 45) “experienced an average of 59.4 and 56.6 days with medical visits per-patient-per-year (PPPY), respectively, including 14.1 and 4.6 inpatient days.” [35] Regardless of pharmaceutical or hospital-use economic impact, families of children receiving motor, speech, and survival benefit from nusinersen speak of the miraculous impact of the medication, which exceeds a describable cost value for those children and families. 

### 4.3. Increase in Worry

An important finding from this study was how worry started at the lowest in the maintenance cohort, but worry notably increased longitudinally. Prior studies have shown worry peak at time of decision making about starting a new medication with unknown outcomes and concern for side effects. While nusinersen has been shown to prolong survival in infants with SMA [19,20,21,22] and improve motor function [23,24], the parents involved in this study engaged in treatment decision making prior to the more recent accumulation of outcomes-based data and thus were venturing into the unknown. 

Guilt regarding genetic diagnoses and uncertainties introduced by new therapies compound the underlying unpredictable trajectory of SMA [36,37], resulting in realistic worry at medication start. Parents of children starting nusinersen report worrying about “making difficult treatment choices” as well as “reactions, side effects, and worsening quality of life” [33,38]. A qualitative study of German parents of children with SMA Type 1 depicted “significant uncertainty and stress among caregivers prior to the actual treatment. Further, concerns persisted that nusinersen could not be approved or that the child could be excluded due to an insufficient treatment response” [26]. While medical teams may consider nusinersen generally well tolerated and efficacious, parents depict worry about their child not responding to nusinersen, requiring treatment interruptions, and experiencing complications [39].

As data show that earlier initiation of treatment is associated with more efficacy on functionality (such as ambulation) [40], family caregivers recognize time-sensitive decision making which may compound the sense of worry or urgency at initiation. Secondary spine and thorax deformities are frequent in children with SMA [41], adding worry for many families about not only the frequency of sedation but also the lumbar puncture itself [42]. Parents weigh the hoped-for benefits of nusinersen with concern about the child’s discomfort. Even for parents of children with SMA who did not experience nusinersen-related adverse events, realities of disease-specific adverse events such as cough, respiratory infections, and weakness continue to cause concern [4]. This study revealed that worry did not dissolve or mitigate with time, but instead seemed to increase longitudinally. This pattern of sustained worry as captured in this study hints at ongoing concern that patients and families have about whether the medication will continue working, whether there will be a delayed side effect, and the extent to which benefit may be sustained. 

### 4.4. Study Strengths and Limitations 

Strengths of this study include access to not only proxy-report but also pediatric patient-reported outcomes, now recognized as the gold standard for drug impact reporting [43]. Additional study strength includes use of quality of life metrics validated for this population and obtainment of surveys at more than one timepoint. Study limitations include single-site enrollment. This study did not control for whether children had missed any doses of nusinersen or adverse event/side effect profile of medication administration. 

## 5. Conclusions

Nusinersen has offered a form of medical hope to children with SMA and their family caregivers with measurable impact on motor function and ambulation, despite the cost and challenges with administration. As the science advances to now include gene therapy, an interim goal would be additional treatment options with less burden on patients for SMA such as oral administration or one-time infusions. The lived experience of children with SMA receiving nusinersen warrants attentiveness towards ways to continually improve their quality of life. This includes consideration of ways to support family emotion and economic burden as well as foster family-centric communication. Future studies would ideally explore the impact of nusinersen and novel pharmaceutical interventions on functional abilities chronologically and longitudinally with correlated quality of life and family impact metrics.

## Figures and Tables

**Table 1:** PedsQL—Family Impact Module.

	Full Cohort (*n* = 30) *			
Variable	Baseline	Follow Up	Difference	*p*-Value
Physical Functioning	54.9 (21.8)	53.6 (20.5)	−1.2 (14.9)	0.65
Emotional Functioning	56.5 (21.5)	60.2 (20.6)	3.7 (14.2)	0.168
Social Functioning	50.4 (23.8)	50.6 (24.7)	0.2 (18.5)	0.951
Cognitive Functioning	56.2 (24.9)	59.6 (22.1)	3.3 (18.3)	0.326
Communication	50.3 (19.8)	54.2 (23.1)	3.9 (17.1)	0.222
Worry	51.3 (19.5)	55.7 (21.0)	4.3 (15.6)	0.138
Daily Activities	33.3 (23.1)	37.2 (29.4)	3.9 (21.9)	0.338
Family Relationship	60.0 (25.2)	63.7 (26.8)	3.7 (22.4)	0.377
PedsQL Family Impact Total Score	52.7 (18.9)	55.3 (19.5)	2.5 (11.0)	0.218
Parent HRQL Summary Score	54.6 (19.8)	56.0 (19.5)	1.3 (12.7)	0.574
Family Functioning Score	50.0 (22.3)	53.8 (26.5)	3.8 (19.6)	0.304

* Reported mean (SD); *p*-value from paired *t*-tests.

**Table 2:** Child Self-Repot PedsQL.

	Full Cohort (*n* = 16) *			
Variable	Baseline	Follow Up	Difference	*p*-Value
Neuromuscular Disease	56.7 (17.7)	56.2 (17.4)	−0.5 (12.0)	0.865
Communication	60.4 (40.4)	66.7 (30.5)	7.6 (23.4)	0.308
Family Resources	60.4 (25.7)	70.7 (18.0)	9.5 (18.0)	0.108
Total	58.2 (17.6)	59.9 (14.9)	1.7 (9.9)	0.497

* Reported as mean (SD); *p*-values are from paired *t*-tests.

**Table 3:** Proxy -Report (Family Caregiver) PedsQL.

	Full Cohort (*n* = 30) *			
Variable	Baseline	Follow Up	Difference	*p*-Value
Neuromuscular Disease	54.9 (16.5)	53.8 (15.7)	−1.1 (12.1)	0.625
Communication	43.9 (38.1)	48.1 (37.1)	4.2 (17.5)	0.202
Family Resources	50.8 (22.7)	55.3 (18.8)	4.5 (17.1)	0.16
Total	52.7 (17.4)	53.3 (16.2)	0.6 (10.6)	0.755

* Reported as mean (SD); *p*-values are from paired t-tests.

**Table 4:** Significant Difference in Quality of Life Scales by Cohort.

Module	Domain	Cohort	Baseline	Follow Up	Difference	*p*-Value
Child-PedsQL	Worry	SMA 1, *n* = 2	36.7 (15.3)	71.2 (11.8)	31.7 (2.9)	0.003
Child-PedsQL	Total Quality of Life	SMA 3, *n =* 3	68.4 (13.1)	73.0 (12.8)	4.6 (2.2)	0.068
Parent-PedsQL	Communication	SMA 3, *n =* 4	52.1 (25.8)	64.6 (31.5)	12.5 (8.3)	0.058
Parent-PedsQL	Family Resources	SMA 3, *n =* 4	58.8 (12.5)	68.8 (15.5)	10.0 (7.1)	0.066
Family Impact	Worry	SMA 1, *n =* 13	45.0 (12.7)	50.8 (12.6)	5.8 (9.8)	0.054
Family Impact	Daily Activities	SMA 2, *n =* 13	39.1 (28.1)	52.6 (32.5)	13.5 (23.9)	0.065

SMA 1, 2, and 3 reflect diagnostic subtype. colors are useful to reveal statistical significance reached vs. close.

**Table 5:** Significant Difference in Quality of Life Scales by Timeframe.

Module	Domain	Cohort	Baseline	Follow Up	Difference	*p*-Value
Child-PedsQL	Family Resources	L-M, *n =* 7	52.5 (26.4)	71.7 (15.4)	23.0 (12.5)	0.015
Parent-PedsQL	Communication	0 or L, *n =* 19	45.2 (34.5)	53.9 (33.7)	8.8 (17.2)	0.04
Parent-PedsQL	Communication	0-M, *n =* 8	53.1 (34.5)	67.7 (32.6)	14.6 (20.8)	0.087
Parent-PedsQL	Communication	M-M, *n =* 11	37.9 (42.1)	−3.8 (15.5)	0.437	0.056
Family Impact	Emotional Functioning	0 or L, *n =* 19	60.0 (16.2)	64.7 (15.6)	4.7 (9.9)	0.052
Family Impact	Emotional Functioning	0-M, *n =* 8	56.2 (7.5)	65.4 (15.3)	7.5 (2.9)	0.014
Family Impact	Emotional Functioning	M-M, *n =* 11	53.0 (16.7)	57.1 (28.5)	8.0 (12.7)	0.078
Family Impact	Communication	0-M, *n =* 8	54.2 (10.8)	55.6 (26.7)	8.3 (6.8)	0.092
Family Impact	Communication	M-M, *n =* 11	43.3 (19.6)	54.2 (25.0)	13.3 (17.7)	0.041
Family Impact	Worry	M-M, *n =* 11	44.5 (16.1)	57.9 (23.2)	10.5 (15.2)	0.056
Family Impact	HRQL Summary Score	M-M, *n =* 11	47.0 (12.2)	52.7 (26.6)	7.2 (11.6)	0.08
Family Impact	Family Impact Total Score	M-M, *n =* 11	45.7 (13.0)	52.4 (26.1)	8.6 (10.2)	0.027

Abbreviations—0 = No Nusinersen, L = Loading Phase, and M = Maintenance Dosing.

## Data Availability

Data can be made available upon reasonable request to senior author.
